# Inhibition of LOXL2 Enhances the Radiosensitivity of Castration-Resistant Prostate Cancer Cells Associated with the Reversal of the EMT Process

**DOI:** 10.1155/2019/4012590

**Published:** 2019-01-27

**Authors:** Peng Xie, Hongliang Yu, Feijiang Wang, Feng Yan, Xia He

**Affiliations:** ^1^Department of Radiation Oncology, The Affiliated Cancer Hospital of Nanjing Medical University & Jiangsu Cancer Hospital & Jiangsu Institute of Cancer Research, China; ^2^Department of Clinical Laboratory, The Affiliated Cancer Hospital of Nanjing Medical University & Jiangsu Cancer Hospital & Jiangsu Institute of Cancer Research, China

## Abstract

**Introduction:**

Radiotherapy is the mainstay in the treatment of prostate cancer. However, significant radioresistance of castration-resistant prostate cancer (CRPC) cells constitutes a main obstacle in the treatment of this disease. By using bioinformatic data mining methods, LOXL2 was found to be upregulated in both androgen-independent prostate cancer cell lines and radioresistant tumor samples collected from patients with prostate cancer. We speculate that LOXL2 may play an important role in the radioresistance of CRPC cells.

**Methods:**

The effect of LOXL2 knockdown on the radiosensitivity of androgen-independent prostate cancer cells lines was measured by the clonogenic assay and xenograft tumor experiments under in vitro and in vivo conditions, respectively. In studies on the mechanism, we focused on the EMT phenotype changes and cell apoptosis changes induced by LOXL2 knockdown in DU145 cells. The protein levels of three EMT biomarkers, namely, E-cadherin, vimentin, and N-cadherin, were measured by western blotting and immunohistochemical staining. Cell apoptosis after irradiation was measured by flow cytometry and caspase-3 activity assay. Salvage experiment was also conducted to confirm the possible role of EMT in the radiosensitization effect of LOXL2 knockdown in CRPC cells.

**Results:**

LOXL2 knockdown in CRPC cells enhanced cellular radiosensitivity under both in vitro and in vivo conditions. A significant reversal of EMT was observed in LOXL2-silenced DU145 cells. Cell apoptosis after irradiation was significantly enhanced by LOXL2 knockdown in DU145 cells. Results from the salvage experiment confirmed the key role of EMT process reversal in the radiosensitization effect of LOXL2 knockdown in DU145 cells.

**Conclusions:**

LOXL2 plays an important role in the development of cellular radioresistance in CRPC cells. Targeting LOXL2 may be a rational avenue to overcome radioresistance in CRPC cells. A LOXL2-targeting strategy for CRPC treatment warrants detailed investigation in the future.

## 1. Introduction

Prostate cancer is one of the most common malignancies in men from western countries such as the United States and certain countries in Europe; the incidence of prostate cancer in Asian countries has also been increasing in the past decades [1]. Radiotherapy (RT) plays an important role in the treatment of prostate cancer, thus serving as either a primary radical treatment or an adjuvant therapy after radical prostatectomy or hormone castration regimen. The effectiveness of RT has been well established in the past decades [2]. However, when primary prostate cancer proceeds to the castration-resistant prostate cancer (CRPC) stage, the tumor shows substantial resistance to most conventional therapies including RT [3, 4]. Thus, the radioresistance of CRPC constitutes an important impediment to RT in curing patients of prostate cancer.

The main cellular function of lysyl oxidase-like 2 (LOXL2), a member of the lysyl oxidase (LOX) family, was reported to promote the crosslinking of collagen and elastin in the extracellular matrix (ECM) [5]. Recently, more attention in cancer research was given to its role in the regulation of extracellular and intracellular cell signaling pathways. Aberrant expression of LOXL2 was often associated with elevated metastasis potency of tumor cells, and the outcome was reported as a poor prognosis in various kinds of malignancies including gastric cancer, head and neck squamous cancer, and breast cancer [6–8]. However, a rare study that focused on the role of LOXL2 in prostate cancer is available. Its expression profile and biochemical role in castration evolution as well as the radiosensitivity of prostate cancer cells were largely unknown.

In the present study, we investigated differences in the expression of LOXL2 between androgen-dependent and -independent prostate cancer cell lines and the regulating effect of LOXL2 on the radiosensitivity of CRPC cells. Our results revealed that the LOXL2 level was elevated in CRPC cells and tightly associated with the radiosensitivity of CRPC cells. Inhibition of LOXL2 in DU145 cells could significantly enhance cellular radiosensitivity. On investigating the mechanism, we found that the regulation effect of LOXL2 on cellular radiosensitivity is attributed mainly to the effect on cellular epithelial-mesenchymal transition (EMT) phenotype. To the best of our knowledge, this is the first study that focuses on the radiosensitivity regulation effect of LOXL2 in cancer cells, although we focused mainly on CRPC cells.

## 2. Materials and Methods

### 2.1. Cell Lines and Cell Culture

DU145, PC3, 22Rv1, and LNCaP prostate carcinoma cell lines were obtained from the Cell Bank of the Chinese Academy of Sciences (Shanghai, China) where they were characterized by mycoplasma detection and short tandem repeat detection. Cells were maintained in RPMI 1640 medium (M&C Gene Technology, Beijing, China) supplemented with 10% fetal bovine serum (FBS; Gibco, Auckland, New Zealand) at 37°C in humidified air containing 5% carbon dioxide. The cells that reached the logarithmic phase were selected for further experiments.

### 2.2. TCGA and GEO Data Download and Bioinformatic Processing

Gene count data from prostate cancer TCGA samples (RNA sequencing) were downloaded from the Genomic Data Commons Data Portal (https://portal.gdc.cancer.gov/). Microarray gene expression profiles of prostate cancer cell lines from the GSE4016 dataset were downloaded from the GEO database (https://www.ncbi.nlm.nih.gov/geo/). To identify the key genes in radioresistant (RR) and radiosensitive (RS) prostate cancer samples, in this study, we defined patients to be RS if they received external radiation over the primary tumor and no recurrence occurred and we defined patients to be RR if recurrence occurred after RT. To identify differentially expressed genes (DEGs), raw data from TCGA and GEO databases were entered into DESeq and limma packages in R, respectively [9, 10]. The definition of DEGs in this study is a difference significant at a p value of <0.05 between two subsets. The intersection genes from the two datasets were then obtained.

### 2.3. Gene Knockdown with Small Interfering RNA (siRNA)

For mRNA knockdown, the following siRNA sequences were validated and used: LOXL2-targeting siRNA (Si-LOXL2) sequences, 5′-GACUGCCAGUGGAUUGAUATT-3′; E-cadherin-targeting siRNA (Si-Ecad) sequences, 5′-UAUGGUCUUGGAGCUUGAUTT-3′; and negative control siRNA (Scramble) sequences, 5′-GGUGUGCUGUUUGGAGGUCTT-3′. All sequences were designed and synthesized by Shanghai GenePharma Co., Ltd. (Shanghai, China). For gene knockdown, DU145 and PC3 cells were seeded onto six-well plates at a density of 10^5^ cells/well, and the plates were incubated for 24 h. Cells were then transfected with control siRNA or LOXL2 siRNA and/or E-cadherin siRNA with Lipofectamine 2000 (Thermo Fisher Scientific, Inc.), according to the manufacturer's instructions. Gene silencing was validated by assessing their mRNA and protein expression levels in DU145 and PC3 cells 48 h after transfection.

### 2.4. LOXL2-Knockdown Stable Cells and Xenograft-Bearing Nude Mice Experiment

LOXL2-targeting shRNA- and scramble-targeting shRNA-expressed lentivirus vectors were designed and synthesized by Shanghai GenePharma Co., Ltd. (Shanghai, China). The targeting sequence of LOXL2 was 5′-GTATGACAACAGCCTCAAG-3′. Selection was performed for 3 days with 800 ng/ml of puromycin until the parental cells in parallel experiments completely died. Tumor irradiation was performed as follows: when the diameter of xenograft tumors reached 6–8 mm, the xenograft mice were irradiated with a single dose (6 Gy). After irradiation, the tumor growth was monitored for up to 3 weeks. Tumors were harvested for further investigation. The tumor volume was calculated using the formula V = *π*/6  (a*∗*b^2^), where a is the longest perpendicular tumor axis and b is the shortest perpendicular tumor axis.

### 2.5. Real-Time Quantitative PCR

Total RNA was isolated from cells using TRIzol Reagent (Thermo) according to the manufacturer's protocol, and cDNA was generated using iScript reagents (Bio-Rad, Hercules, CA). Real-time quantitative PCR based on SYBR-Green gene expression was performed on a StepOne thermocycler (Applied Biosystems, Foster City, CA). Normalization to GAPDH mRNA was done, and fold change in the gene mRNA level was calculated by the ΔΔCt method. For quantitative PCR, the following primers were used: GAPDH-forward, 5′-AACATCATCCCTGCTTCCAC-3′, GAPDH-reverse, 5′-GACCACCTGGTCCTCAGTGT-3′, LOXL2-forward, 5′-CACACATACACGTGCACACA-3′, LOXL2-reverse, 5′-AGGCTCTCCCCAAGGAAAT-3′, E-cadherin-forward, 5′-TGCCCAGAAAATGAAAAAGG-3′, E-cadherin-reverse, 5′-GTGTATGTGGCAATGCGTTC-3′.

### 2.6. Western Blotting

The protein expression pattern in LNCaP, 22Rv1, PC3, and DU145 cells was evaluated by western blotting. Briefly, cells were seeded in six-well plates and cultured to 50% confluence. The cells were then transfected with the corresponding siRNA, and the plate was incubated for an additional 48 h. Following the indicated treatments, cell protein extracts were prepared. Western blotting was conducted with 100 *μ*g of the protein extract as described elsewhere [11].

### 2.7. Apoptosis Analysis by Annexin V-FITC/PI Double Staining and Caspase-3 Activity Measurement

The Annexin V-FITC/PI double staining was performed according to the manufacturer's protocol (Beyotime Biotechnology, Jiangsu, China) to measure cell apoptosis. Briefly, 24 h after irradiation, the assigned groups of cells were harvested and processed for Annexin V-FITC/PI staining to detect apoptotic cells; the percentage of apoptotic cells was quantified by flow cytometry. For capsase-3 activity detection, the assigned groups of cells were transfected with corresponding si-RNAs and incubated for 48 h before exposure to 4 Gy radiation. Cells were then centrifuged and resuspended in cold lysis buffer. Approximately 15 min later, cells were centrifuged for 10 min to collect the supernatants. Then, 10 *μ*l of the capsase-3 substrate Ac-DEVD-pNA (Beyotime Biotechnology, Jiangsu, China) was added to the supernatant, and this mixture was incubated for another 1 h. Absorbance at 405 nm was measured using a spectrophotometer (Molecular Devices, Sunnyvale, CA) to analyze the activity of caspase-3.

### 2.8. Clonogenic Assay

Radiosensitivity survival curves were generated after the clonogenic assay. After pretreating the cells with the assigned si-RNA transfection, DU145 cells were cultured for an additional 48 h. Some of the cells were used for silence efficiency validation and the rest were exposed to different dosages of radiation (0, 2, 4, 6, and 8 Gy); then, they were seeded in six-well plates and grown in quiescence for 14 days. At the end of 14 days, the cells were fixed with methanol and stained with crystal violet, and colonies containing more than 50 cells were counted. After survival fractions at all dosages were calculated, a multitarget single-hit model was applied for fitting data by the method previously described in [11].

### 2.9. Statistical Analysis

Statistical analyses were carried out using GraphPad Prism software, and statistical significance was determined by ANOVA or two-tailed Student's t-test. For all statistical analyses, a p value less than 0.05 was considered as statistically significant.

### 2.10. Ethics Statement

The animal study was carried out in strict accordance with the recommendations in the Guide for the Care and Use of Laboratory Animals of the National Institutes of Health. All efforts were made to minimize suffering. All procedures for cell and animal experiments as well as immunohistochemistry of tissue sections were approved by the Ethics Committee of Jiangsu Cancer Hospital.

## 3. Results

### 3.1. LOXL2 Was Upregulated in CRPC Cell Lines and Radioresistant Prostate Cancer Samples

**Figure 1:**
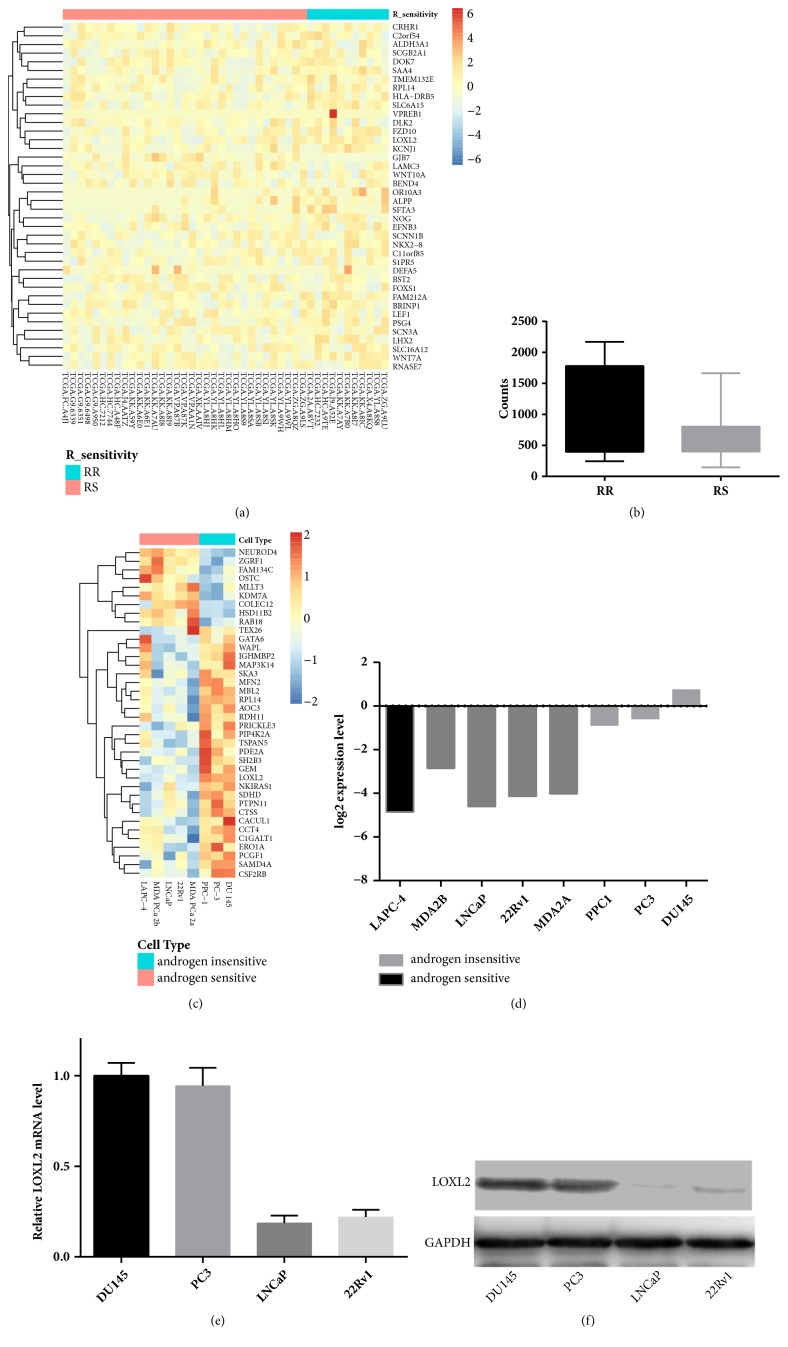
LOXL2 was upregulated in CRPC cell lines and radioresistant prostate cancer samples. (a) Diagrammatic representation of heatmap of DEGs in the TCGA dataset. (b) LOXL2 expression was significantly higher in samples of the radioresistant group (RR) than in the radiosensitive group (RS) from the TCGA dataset. (c) Diagrammatic representation of heatmap of DEGs in prostate cell lines with different androgen dependency status from the GEO dataset. (d) A captured histogram picture from the GEO dataset, which shows that the expression of LOXL2 was upregulated in androgen-independent prostate cell lines. (e) LOXL2 expression was higher in CRPC cells of DU145 and PC3 cells than in androgen-dependent LNCaP and 22Rv1 cells at the mRNA level measured by real-time PCR. (f) LOXL2 expression was higher in DU145 and PC3 cells than in LNCaP and 22Rv1 cells at the protein level. *∗* denotes p <0.05 by Student's t-test.

In the GEO dataset GSE4016, gene expression profiles of eight cell lines with different androgen-dependent status were available. Androgen-dependent cell lines include LAPC-4, MDA PCa 2b, LNCaP, 22Rv1, and MDA PCa 2a, whereas androgen-independent cell lines include PPC-1, PC-3, and DU145. A pool of 189 DEGs was identified in CRPC cell lines as compared with androgen-dependent cell lines. In the TCGA dataset, RNA sequencing data for 551 patients were downloaded. For the missing information about the clinical follow-up of 53 patients, a merged table consisting of 498 samples was formed and contained both RNA sequence information and adaptive clinical information. In these samples, 44 samples were used as the data source for identifying DEGs responsible for radiosensitivity because these samples satisfied the criteria of both a history of external radiation therapy and a clear response outcome. After bioinformatic processing with DESeq package in R, we finally found a pool of 129 DEGs in the TCGA dataset. Within the intersection DEGs of the two datasets, we observed that LOXL2 was consistently upregulated in CRPC cell lines, as determined in the GEO dataset, and radioresistant prostate cancer samples, as determined in the TCGA dataset. We also confirmed this pattern of expression with our own data that LOXL2 was downregulated at both mRNA and protein levels in androgen-dependent LNCaP and 22Rv1 cell lines rather than in DU145 and PC3 cells. All these results are shown in Figure 1.

### 3.2. Knockdown of LOXL2 Radiosensitizes DU145 and PC3 Cells

**Figure 2:**
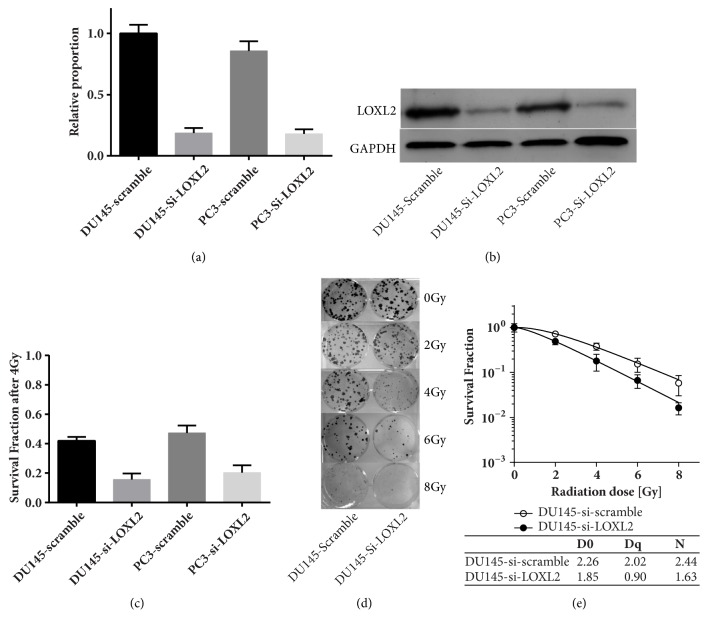
Knockdown of LOXL2 radiosensitizes DU145 and PC3 cells. (a-b) LOXL2 was significantly silenced in DU145 and PC3 cells by si-LOXL2 transfection at both mRNA and protein levels. (c) The radiosensitization effect of LOXL2 knockdown in DU145 and PC3 cells was determined by the clonogenic assay after 4 Gy of irradiation. (d-e) Dose-survival curve derived from the results of clonogenic assay for DU145 cells; the radiobiological parameters D0, Dq, and N are also listed. *∗* denotes p <0.05 by Student's t-test.

As shown in Figures 2(a) and 2(b), we confirmed a significant downregulation of LOXL2 by siRNA in DU145 and PC3 cells at both mRNA and protein levels. Survival fractions after 4 Gy of irradiation were significantly lower in LOXL2 knockdown PC3 and DU145 cells, which were presented in Figure 2(c). The dose-survival curve evaluating the radiosensitization effect of LOXL2 knockdown on DU145 cells was presented in Figures 2(d) and 2(e). We found that knockdown of LOXL2 can significantly radiosensitize PC3 and DU145 cells.

### 3.3. The Radiosensitizing Effect of LOXL2 Knockdown in DU145 Cells Was Associated with the Reversal of EMT Phenotype and an Increase in Cell Apoptosis

**Figure 3:**
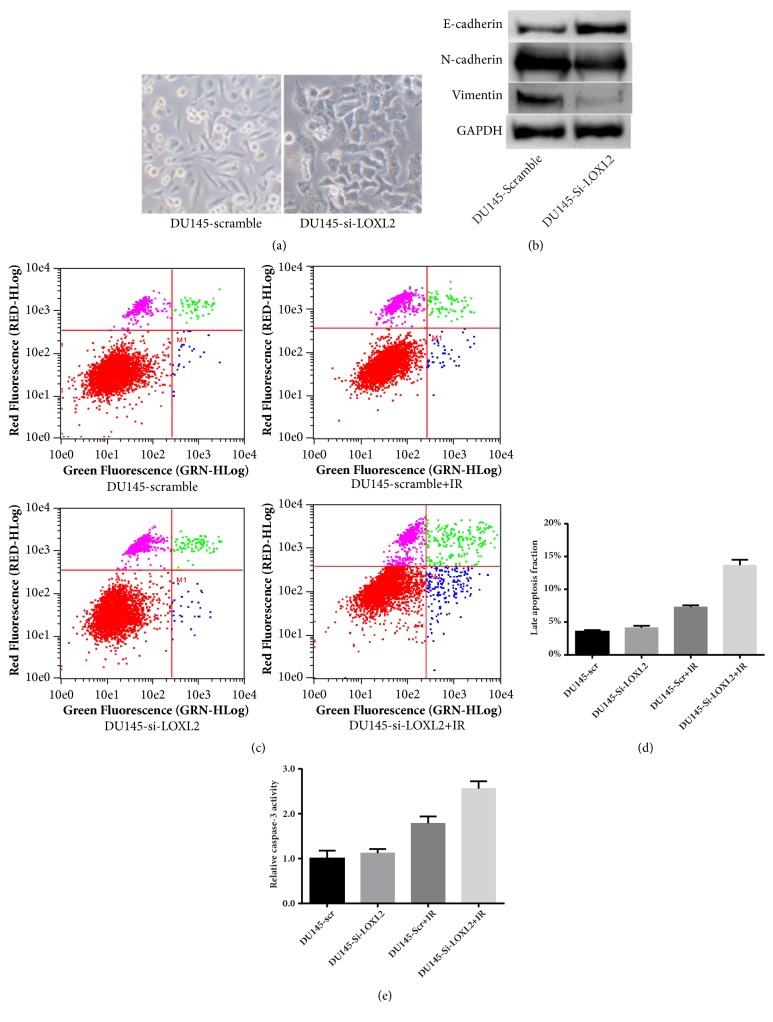
The radiosensitizing effect of LOXL2 knockdown in DU145 cells was associated with the reversal of the EMT phenotype and an increase in cell apoptosis. (a) The typical morphology of DU145 and LOXL2-silenced DU145 cells. (b) Protein expression of three classical EMT biomarkers, namely, E-cadherin, vimentin, and N-cadherin, was determined by western blotting. A significant reversal of the EMT process was observed in LOXL2-knockdown DU145 cells. (c-d) LOXL2 knockdown significantly enhanced cell apoptosis in DU145 cells after a 4 Gy dose of irradiation as measured by Annexin V-FITC/PI double staining. (e) LOXL2 knockdown significantly enhanced cell apoptosis in DU145 cells after a 4 Gy dose of irradiation as measured by the caspase-3 activity assay. *∗* denotes p <0.05 by Student's t-test.

The typical morphology of LOXL2 silenced DU145 cells and control was presented in Figure 3(a). We observed an evident cellular morphology transition when LOXL2 was knocked down in DU145 cells, from an irregular, spindle-like morphology to a wide, flat epithelial appearance, and significant cell-cell adhesion. As shown in Figure 3(b), we studied the changes in the protein expression pattern of three classical EMT biomarkers of E-cadherin, vimentin, and N-cadherin for LOXL2 knockdown in DU145 cells. E-cadherin was significantly upregulated, whereas vimentin and N-cadherin were significantly downregulated when LOXL2 was silenced in DU145 cells. All these results indicate that the mesenchymal phenotype of CRPC DU145 cells was significantly ameliorated, and an epithelial-like phenotype was restored. The EMT process in DU145 cells was significantly reversed. Furthermore, we studied the effect of LOXL2 knockdown on cell apoptosis after irradiation in DU145 cells. As shown in Figure 3(c), the proportion of apoptotic cells after 4 Gy irradiation was significantly increased when LOXL2 expression was silenced. Similarly, as shown in Figure 3(d), as a biomarker of apoptosis, caspase-3 activity was significantly elevated after irradiation at a dose of 4 Gy in LOXL2-silenced DU145 cells as compared with control cells.

### 3.4. The Impact of LOXL2 Knockdown on the Growth of Irradiated Xenograft Tumors

**Figure 4:**
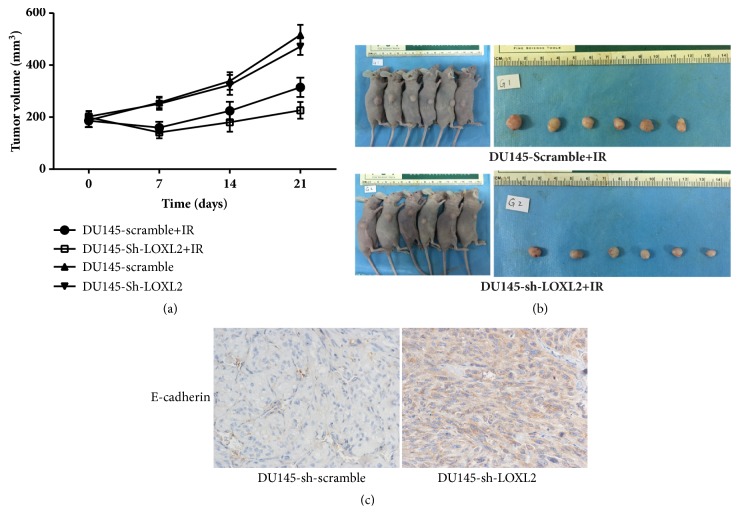
Xenograft-based evaluation of the radiosensitization effect of LOXL2 knockdown in CRPC DU145 cells in vivo. (a) The time course of growth of DU145-scramble and DU145-si-LOXL2 xenograft tumors with or without a local 6 Gy dose of IR treatment. (b) Representative photo of residual tumor of DU145-scramble and DU145-si-LOXL2 cells after a 6 Gy dose of irradiation. (c) IHC staining showed an elevated E-cadherin expression in LOXL2-knockdown xenograft tumor, which represents the reversal of the EMT process in LOXL2-knockdown cells in vivo. *∗* denotes p <0.05 by Student's t-test.

We then assessed whether LOXL2 knockdown-mediated radiosensitization effect on DU145 cells was also applicable in xenograft tumors. Two xenograft models were established by subcutaneously implanting DU145-sh-LOXL2 and DU145-sh-scramble control cells, separately, in nude mice. As shown in Figure 4(a), we found that both xenotransplant tumors grew fast without showing a significant difference in tumor volume between the two groups, namely, the sh-LOXL2 group and the control sh-scramble group, when not irradiated. However, when the two groups of xenograft tumors were subjected to local radiation at a single dose of 6 Gy, we found that the sh-LOXL2 group of mice were more sensitive to treatment, with a significant shrinkage in tumor volume in the sh-LOXL2 group as compared with that in the sh-scramble group, as shown in Figure 4(b). Additionally, results of immunohistochemical staining analysis presented in Figure 4(c) indicated that the EMT biomarker E-cadherin expression in the sh-LOXL2 group was significantly lower than that in the control sh-scramble group. These results disclose the radiosensitization effect of LOXL2 knockdown on xenograft tumors, thus confirming the hypothesis derived from cell-based experiments that the radiosensitization effect of LOXL2 knockdown was attributed to the reversal of the EMT process in DU145 cells.

### 3.5. Salvage Experiment Confirmed EMT Reversion Dominated the Mechanism for Restoring Radiosensitivity in DU145 Cells after LOXL2 Knockdown

**Figure 5:**
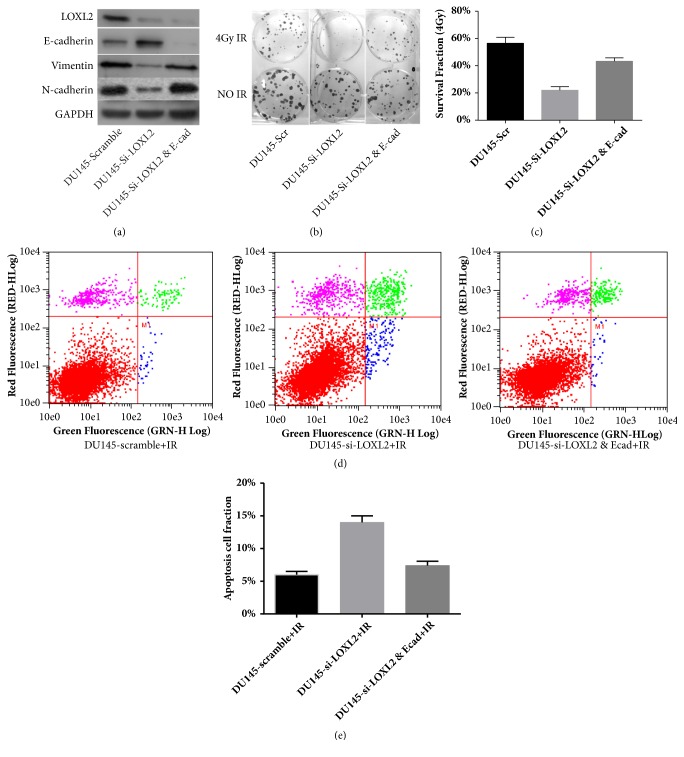
Salvage experiment confirmed that EMT process reversal dominated the mechanism for restoring radiosensitivity in DU145 cells after LOXL2 knockdown. (a) EMT phenotype was coercively restored by simultaneous E-cadherin knockdown in LOXL2-silenced DU145 cells, as represented by the restoration of the expression of vimentin and N-cadherin. (b, c) Coercively restoring the EMT process in LOXL2-silenced DU145 cells restored cellular radioresistance as determined by the clonogenic assay. (d-e) Coercively inducing the EMT process restored cellular radioresistance as determined by cell apoptosis study by Annexin V-FITC/PI double staining. *∗* denotes p <0.05 by Student's t-test.

E-cadherin expression downregulation is not only a manifestation of the EMT process but also a leading cause of EMT phenotype [12, 13]. In salvage experiment, we mandatorily inhibited the E-cadherin expression in LOXL2-silenced DU145 cells by si-E-cad, for coercively inducing EMT phenotype. The results shown in Figure 5(a) demonstrate that vimentin and N-cadherin were upregulated, thus indicating that EMT phenotype was coercively restored in LOXL2-silenced DU145 cells. For evaluating the effect of EMT reversal on restoring radiosensitivity in LOXL2-knockdown DU145 cells, survival fraction of cells at a dose of 4 Gy irradiation (SF4) was obtained. As presented in Figures 5(b) and 5(c), our results showed that SF4 was significantly lower in LOXL2-knockdown DU145 cells than in control cells, thus indicating that DU145 cells were significantly radiosensitized by LOXL2 knockdown. When we mandatorily restored the EMT phenotype by simultaneously inhibiting E-cadherin, SF4 was significantly elevated, and a radioresistance phenotype was restored. Similarly, we found this variation tendency in the cell apoptosis assay as shown in Figures 5(d) and 5(e). Our results showed that knockdown of LOXL2 expression ameliorated the EMT phenotype in DU145 cells and enhanced cellular radiosensitivity, whereas by coercively restoring EMT phenotype by simultaneously inhibiting E-cadherin expression, radioresistance was restored in LOXL2-knockdown DU145 cells. Therefore, reversal of the EMT process plays a dominant role in the radiosensitization effect of LOXL2 knockdown in DU145 cells.

## 4. Discussion

Under certain clinical circumstances, RT is an attractive approach for the treatment of prostate cancer, thus serving as either a primary radical treatment or an adjuvant therapy. Unfortunately, existence of heterogeneity among tumor cells may nourish radioresistance, which impedes the efficacy of RT. Lines of evidence from recent studies suggest that an increase in the radiosensitivity of cancer cells may afford a promising avenue to overcome radioresistance of carcinomas including CRPC [11, 14, 15]. In the present study, we showed for the first time that inhibition of LOXL2 radiosensitized CRPC cells mainly because of the reversal of the EMT process in cells. To the best of our knowledge, this is the first study that focuses on the role of LOXL2 in radiosensitivity in cancer cells.

LOXL2, a member of the LOX family, exerts its biochemical function mainly in the scenario of ECM. It catalyzes oxidative deamination of peptidyl lysine on collagen and elastin, thereby generating a highly reactive aldehyde group to initiate intermolecular crosslinking [16]. It gained increasing attention in recent years for the discovery of its aberrant expression and poor prognosis implication in many kinds of malignancy [7, 17–19]. Importantly, owing to its increasing crucial role discovered in recent years, researchers studied LOXL2 in depth and successfully delineated the elegant structure of this protein in anatomic resolution; this will greatly facilitate the structure-function study and targeted drug development [16]. We selected LOXL2 for investigation by a bioinformatic approach. Prostate cancer showed substantial treatment resistance when this malignancy progressed from an androgen-dependent state to the CRPC state. The molecular differences between CRPC cells and androgen-dependent tumor cells may comprise the underlying mechanisms for radioresistance of CRPC cells. By data mining of high-throughput microarray data of prostate cancer cell lines deposited in the GEO database, LOXL2 was found to be significantly upregulated in all three CRPC cell lines as compared with the androgen-dependent prostate cancer cell lines. Additionally, LOXL2 was again found to be significantly upregulated in RR samples in the TCGA dataset. Therefore, we speculated that LOXL2 may play an important role in the development of cellular radioresistance in CRPC cells.

The present study confirmed that LOXL2 plays a key role in radioresistance of DU145 cells. Inhibition of LOXL2 expression in DU145 cells significantly enhanced the radiosensitivity of DU145 cells. We found that the main mechanism for exhibiting the radiosensitization effect resulting from LOXL2 inhibition may rely on the reversal of the EMT process in DU145 cells. We focused on the EMT process for studying the mechanism for the following logistic reasons: first, LOXL2 mainly exerts its physiological function in the ECM, while the main characteristic of the EMT process was that epithelial cells lose their polarized organization and cell-cell junctions [13, 20]; second, we observed an evident cellular morphology transition when LOXL2 was knocked down in DU145 cells, from an irregular, spindle-like morphology to a wide, flat epithelial appearance, and significant cell-cell adhesion; finally, LOXL2 was reported to be closely related to E-cadherin expression and the EMT process [17, 21], and EMT was a well-known leading cause of cellular radioresistance [13]. Naturally, studies on mechanism and salvage experiments confirmed the mainstay effect of the reversal of the EMT process in the radiosensitization effect of LOXL2 knockdown in DU145 cells.

Our data are in line with those given in other studies that highlight the contribution of the EMT process to the induction of cellular radioresistance. Nevertheless, further experiments are necessary to elucidate the underlying mechanisms of the regulation effect of LOXL2 on E-cadherin expression and EMT phenotype in this specific context in CRPC. This regulation effect may be linked to various mechanisms, including LOXL2 collaboration with the transcription factor E47 as a regulator of SNAI1, thereby affecting the induction of EMT [17]; LOXL2 was directly regulated by the miR-200/ZEB1 axis, thereby contributing to the induction of EMT [19]; other evidence showed that LOXL2 was required and adequate for hypoxic repression of E-cadherin, which mediates the cellular EMT process [21]. As cancers are heterogeneous, we speculate that these mechanisms may contribute differently in different tumor microenvironments. So, for the possible future application of LOXL2 as a druggable target in the treatment of CRPC, the detailed mechanisms underlying the EMT reversion caused by LOXL2 inhibition still warrant more studies in future.

In summary, we determined that LOXL2 was upregulated in both CRPC cell lines and RR prostate cancer samples by a bioinformatic approach and confirmed this with our own real-time quantitative PCR data. Knockdown of LOXL2 in CRPC cells significantly enhances cellular radiosensitivity. The main underlying mechanism of the radiosensitization effect of LOXL2 knockdown may be attributed to the reversal of the EMT process. On the basis of our results, we postulate that targeting LOXL2 may be a rational avenue to overcome treatment resistance including radioresistance of CRPC cells. A LOXL2-targeting strategy for the treatment of CRPC warrants more investigation in the future.

## Data Availability

Gene count data from prostate cancer TCGA samples were downloaded from the Genomic Data Commons Data Portal (https://portal.gdc.cancer.gov/). Microarray gene expression profiles of prostate cancer cell lines from the GSE4016 dataset were downloaded from the GEO database (https://www.ncbi.nlm.nih.gov/geo/). Other data and materials supporting the conclusions of this article are included within the article.

## References

[1] Torre L. A., Bray F., Siegel R. L., Ferlay J., Lortet-Tieulent J. (2015). Global cancer statistics, 2012. *CA: A Cancer Journal for Clinicians*.

[2] Hamdy F. C., Donovan J. L., Lane J. A. (2016). 10-Year outcomes after monitoring, surgery, or radiotherapy for localized prostate cancer. *The New England Journal of Medicine*.

[3] Nakfoor B., Zietman A., Gerweck L., Shipley W. (1994). An assessment of the in vitro radiosensitivity of a human prostate cancer cell line: The influence of the hormonal millieu. *International Journal of Radiation Oncology Biology Physics*.

[4] Wade C. A., Kyprianou N. (2018). Profiling prostate cancer therapeutic resistance. *International Journal of Molecular Sciences*.

[5] Kim Y.-M., Kim E.-C., Kim Y. (2011). The human lysyl oxidase-like 2 protein functions as an amine oxidase toward collagen and elastin. *Molecular Biology Reports*.

[6] Kasashima H., Yashiro M., Kinoshita H. (2014). Lysyl oxidase-like 2 (LOXL2) from stromal fibroblasts stimulates the progression of gastric cancer. *Cancer Letters*.

[7] Ahn S. G., Dong S. M., Oshima A. (2013). LOXL2 expression is associated with invasiveness and negatively influences survival in breast cancer patients. *Breast Cancer Research and Treatment*.

[8] Peinado H., Moreno-Bueno G., Hardisson D. (2008). Lysyl oxidase-like 2 as a new poor prognosis marker of squamous cell carcinomas. *Cancer Research*.

[9] Anders S. (2010). *Analysing RNA-Seq data with the DESeq package*.

[10] Ritchie M. E., Phipson B., Wu D. (2015). *limma* powers differential expression analyses for RNA-sequencing and microarray studies. *Nucleic Acids Research*.

[11] Yu H., Li X., Sun S., Gao X., Zhou D. (2012). C-Met inhibitor SU11274 enhances the response of the prostate cancer cell line DU145 to ionizing radiation. *Biochemical and Biophysical Research Communications*.

[12] Liang X., Xu X., Wang F. (2015). E-cadherin knockdown increases *β*-catenin reducing colorectal cancer chemosensitivity only in three-dimensional cultures. *International Journal of Oncology*.

[13] Theys J., Jutten B., Habets R. (2011). E-Cadherin loss associated with EMT promotes radioresistance in human tumor cells. *Radiotherapy & Oncology*.

[14] Ferlazzo M. L., Bourguignon M., Foray N. (2017). Functional Assays for Individual Radiosensitivity: A Critical Review. *Seminars in Radiation Oncology*.

[15] Ahmed K. A., Caudell J. J., El-Haddad G. (2016). Radiosensitivity differences between liver metastases based on primary histology suggest implications for clinical outcomes after stereotactic body radiation therapy. *International Journal of Radiation Oncology Biology Physics*.

[16] Zhang X., Wang Q., Wu J., Wang J., Shi Y., Liu M. (2018). Crystal structure of human lysyl oxidase-like 2 (hLOXL2) in a precursor state. *Proceedings of the National Acadamy of Sciences of the United States of America*.

[17] Canesin G., Cuevas E. P., Santos V. (2015). Lysyl oxidase-like 2 (LOXL2) and E47 EMT factor: Novel partners in E-cadherin repression and early metastasis colonization. *Oncogene*.

[18] Kato M., Kurozumi A., Goto Y. (2017). Regulation of metastasis-promoting LOXL2 gene expression by antitumor microRNAs in prostate cancer. *Journal of Human Genetics*.

[19] Peng D. H., Ungewiss C., Tong P. (2017). ZEB1 induces LOXL2-mediated collagen stabilization and deposition in the extracellular matrix to drive lung cancer invasion and metastasis. *Oncogene*.

[20] Stark T. W., Hensley P. J., Spear A., Pu H., Strup S. S., Kyprianou N. (2017). Predictive value of epithelial-mesenchymal-transition (EMT) signature and PARP-1 in prostate cancer radioresistance. *The Prostate*.

[21] Schietke R., Warnecke C., Wacker I. (2010). The lysyl oxidases LOX and LOXL2 are necessary and sufficient to repress E-cadherin in Hypoxia: Insights into cellular transformation processes mediated by HIF-1. *The Journal of Biological Chemistry*.

